# PROPER I: frequency and appropriateness of psychotropic drugs use in nursing home patients and its associations: a study protocol

**DOI:** 10.1186/1471-244X-13-307

**Published:** 2013-11-15

**Authors:** Klaas van der Spek, Debby L Gerritsen, Martin Smalbrugge, Marjorie HJMG Nelissen-Vrancken, Roland B Wetzels, Claudia HW Smeets, Sytse U Zuidema, Raymond TCM Koopmans

**Affiliations:** 1 Department of Primary and Community Care, Centre for Family Medicine, Geriatric Care and Public Health, Radboudumc, Huispost 117 ELG, P.O. Box 9101, 6500 HB Nijmegen, The Netherlands; 2 Department of General Practice and Elderly Care Medicine/EMGO + Institute for Health and Care Research, VU Medical Center, P.O. Box 7057, 1007 MB Amsterdam, The Netherlands; 3 Dutch Institute for Rational Use of Medicine, P.O. Box 3089, 3502 GB Utrecht, The Netherlands; 4 Department of General Practice, University of Groningen, University Medical Center Groningen, HPC FA21, PO Box 196, 9700 AD Groningen, The Netherlands

**Keywords:** Nursing home, Dementia, Neuropsychiatric symptoms, Psychotropic drug use, Environment

## Abstract

**Background:**

Nursing home patients with dementia use psychotropic drugs longer and more frequently than recommended by guidelines implying psychotropic drugs are not always prescribed appropriately. These drugs can have many side effects and effectiveness is limited. Psychotropic drug use between nursing home units varies and is not solely related to the severity of neuropsychiatric symptoms. There is growing evidence indicating that psychotropic drug use is associated with environmental factors, suggesting that the prescription of psychotropic drugs is not only related to (objective) patient factors. However, other factors related to the patient, elderly care physician, nurse and the physical environment are only partially identified. Using a mixed method of qualitative and quantitative research, this study aims to understand the nature of psychotropic drug use and its underlying factors by identifying: 1) frequency and appropriateness of psychotropic drug use for neuropsychiatric symptoms in nursing home patients with dementia, 2) factors associated with (appropriateness of) psychotropic drug use.

**Methods:**

A cross-sectional mixed methods study. For the quantitative study, patients with dementia (n = 540), nursing staff and elderly care physicians of 36 Dementia Special Care Units of 12 nursing homes throughout the Netherlands will be recruited. Six nursing homes with high average rates and six with low average rates of psychotropic drug use, based on a national survey about frequency of psychotropic drug use on units, will be included. Psychotropic drugs include antipsychotics, anxiolytics, hypnotics, antidepressants, anticonvulsants and anti-dementia drugs. Appropriateness will be measured by an instrument based on the Medication Appropriateness Index and current guidelines for treatment of neuropsychiatric symptoms. Factors associated to psychotropic drug use, related to the patient, elderly care physician, nurse and physical environment, will be explored using multilevel regression analyses. For the qualitative study, in depth interviews with staff will be held and analyzed to identify and explore other unknown factors.

**Discussion:**

This study will provide insight into factors that are associated with the frequency and appropriateness of psychotropic drug use for neuropsychiatric symptoms. Understanding psychotropic drug use and its associations may contribute to better dementia care.

## Background

In the Netherlands approximately 37.000 patients with dementia reside in Dementia Special Care Units (DSCUs) of nursing homes [1,2]. The prevalence of neuropsychiatric symptoms (NPS) associated with dementia is high, more than 80% [3], and frequently a reason for prescription of psychotropic drugs (PDs) [4-6]. However, psychosocial interventions and restraints are also commonly used in the management of NPS [7]. Psychotropic drug use (PDU) rates in institutionalized patients with dementia vary from 63%-75% [6,8,9]. It is also known that antipsychotic use varies among countries between 11% and 52% [6,10-12].

PDs have considerable side effects. Antipsychotics are associated with increased occurrence of extrapyramidal symptoms, somnolence, increased risk for stroke and pneumonia and higher mortality rates [13-15]. Anxiolytic and hypnotic drugs are associated with falls [16]. PDs in general [17] and antipsychotics in particular also have negative effects on quality of life [18].

Long-term or inappropriate use of antipsychotics is common [19], a recent study found that 31% of the nursing home patients used PDs for a sustained period of at least 2 years [9] and in another study 74% of dementia patients in nursing homes used PDs for 83% of their nursing home stay [20]. This does not comply with available evidence on risks, side effects, limited evidence for efficacy of these drugs and long-term inefficacy [15,21,22]. That is why guidelines emphasize the restricted, short-term use and thus the appropriateness of PDU [23].

**Figure 1:**
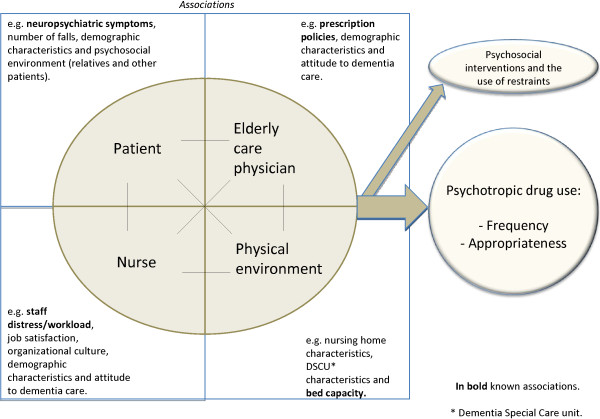
A conceptual framework on psychotropic drug use in nursing homes and its associations.

PDU varies considerably among nursing homes and DSCUs [24,25]. This could partly be explained by different prevalence rates of NPS among patients on DSCUs [3]. However there is growing evidence that this inter-DSCU variation in PDU is not only related to the severity of patients’ NPS [6,26]. The PDU variation is also related to drug prescription policies of the Elderly Care Physician (ECP) [5], staff distress/workload [26], physical environmental factors [25], and the bed capacity of the nursing home [27] (see Figure 1).

Although studies [26,27] investigated frequency of PDU and its associated environmental factors a large proportion, 80%, of the variation in PDU between DSCUs is unexplained [25]. The unexplained variation of PDU, the long-term use and the inter-DSCU variation raise questions not only about appropriateness of prescription, but also about factors associated with the variation in frequency and appropriateness of PDU. That is why we propose a conceptual framework of PDU and four categories of factors with which PDU is hypothesized to be associated: patient, ECP, nurse and physical environment. More specifically, possible other associations related to PDU are: 1. patients’ demographic characteristics and influence of psychosocial environment (relatives and other patients) 2. physicians’ demographic characteristics and attitude to dementia care 3. nurses’ job satisfaction, experienced organizational culture, demographic characteristics and attitude to dementia care 4. the physical environment, e.g. nursing home characteristics and DSCU characteristics.

Depicted in the conceptual framework we hypothesize that PDU frequency and appropriateness are associated with these four categories of factors, the use of psychosocial interventions and restraints are seen as alternatives to PDU in the framework (see Figure 1). To obtain full insight in (possible) associations mixed methods of quantitative and qualitative research will be used.

We aim to study: 1. the frequency and appropriateness of PDU for NPS in nursing home patients with dementia 2. factors associated with frequency and appropriateness of PDU related to patient, ECP, nurse and physical environment.

## Methods/Design

### Design and eligibility

This study, the PROPER I study (PRescription Optimization of Psychotropic drugs in Elderly nuRsing home patients with dementia) is a cross-sectional mixed methods study and will be followed by the PROPER II study [28], a multi-center cluster randomized controlled, pragmatic trial on the efficacy of structured repeated multidisciplinary review on psychotropic drugs. The eligibility of nursing homes is based on a survey among ECPs working in nursing homes that we will carry out among all members of Verenso, the Dutch association of ECPs and community geriatricians. ECPs will be asked to count the number of patients, living on the DSCU they are responsible for, that receive one or more PDs. Nursing homes will be eligible if their ECPs fill in the survey about PDU for at least 3 DSCUs.

### Study population and recruitment

According to our calculations (see section on Sample size), 36 DSCUs need to be recruited. Based on the results of the survey, 36 DSCUs will be divided over six nursing homes with high and six with low DSCU overall PDU rates. DSCUs with medium rates will be accepted if the nursing home’s overall rate is high or low on average; at least two out of three DSCUs need to score high or low within a nursing home. With this selection method the contrast in PDU among nursing homes is increased, which could facilitate finding relevant parameters of PDU, without loss of statistical dispersion for our analyses. No geographical considerations will be made in the recruitment process.

### Measurements

The following instruments will be used to explore frequency and appropriateness of PDU and its associations, i.e. patient, ECP, nurse and physical environment related associations. Associations will be explored by quantitative and qualitative measures.

### Quantitative measures

#### Frequency and appropriateness of PDU, primary outcome

PDU will be classified using the Anatomical Therapeutical Chemical (ATC) classification [29] and grouped into antipsychotics, anxiolytics, hypnotics, antidepressants, anticonvulsants and anti-dementia drugs.

For determining appropriateness of psychotropic drug use a screening tool will be developed, based on the Medication Appropriateness Index (MAI). The MAI was developed in 1992 [30] to determine the drug’s appropriateness for individual patients on 10 items and is proven to be reliable [31] and applicable in the Dutch nursing home setting [32]. However, the MAI is not specifically developed as a tool to screen medical files for appropriateness of prescription of individual psychotropic drugs in dementia and thus does not sufficiently suit the needs for this study. We will therefore adapt the original MAI and develop an instrument that screens medical files for appropriateness of psychotropic drug prescription in dementia. The instrument will primarily screen PDs based on the Dutch association of ECP and community geriatricians (Verenso) guideline for problem behavior [23]. The instrument will also include information about interactions and contraindications that originates from the database of the Royal Dutch Association for the advancement of Pharmacy (KNMP) [33]. PD information that is not provided by the Dutch Verenso guideline, will be derived from ‘Farmacotherapeutisch Kompas’ [34], published by the Dutch Health Care Insurance Board (CVZ) and based on the summary of product characteristics (SPC) [35]. Items will be weighted by an expert panel of pharmacists and ECPs who categorize the relative contribution of each item to the level of drug appropriateness.

### Patient factors

NPS will be assessed with the validated Dutch version of the 12-item Neuropsychiatric Inventory- Questionnaire (NPI-Q) [36,37]. The NPI-Q assesses NPS in dementia and caregiver distress. The NPI-Q measures the occurrence and severity of NPS on a three-point Likert scale and associated caregiver burden on a five-point Likert scale. Additionally, frequency of agitation and aggression will be assessed with the Cohen-Mansfield Aggression Inventory (CMAI) [38], of which the original and the translated Dutch version has been proven reliable and valid [39,40]. The CMAI consists of 29 individual items, each rated at a seven-point Likert scale, combined to 3 subscales of (physically) aggressive, physically non-aggressive and verbally agitated behavior [39]. Information about other patient characteristics that will be derived from patients’ charts are: duration of institutionalization, dementia-type, number of falls, demographic characteristics (date of birth, sex), the use of activities, the use of psychosocial interventions (reality orientation training, reminiscence, validation, aromatherapy, music therapy, light therapy, psychoeducation, sensory activation/snoezelen, multisensory stimulation, cognitive stimulation and psychomotor therapy) and restraints (use of side rails, using a deep chair for patients, use of table stand or chair at table, forced or camouflaged administration of sedative medication, fixing patients with tools (tires, span sheets, tear suits, wristbands, swedish bands), seclude in room with/without the door locked, forced administration of fluid or food and use of electronic alerts).

### ECP factors

‘Attitude to dementia care’ will be measured by Approaches to Dementia Questionnaire (ADQ) [41]. The ADQ consists of 19 items, on a five-point Likert scale and measures hopefulness and person-centredness of professionals in dementia care. Higher scores indicate positive attitudes. The total score ranges from 19–95, the 8-item sub score ‘Hope’ from 8–40, and the 11-item sub score ‘Person-centeredness’ from 11–55. Information about demographic characteristics of the physician/ECP will be collected: age, sex, years of work experience, number of years since education/specialization.

### Nurse factors

Experienced organizational culture will be measured with the Competing Values Framework Scale (CVFS) [42], the validated Dutch version [43], a 6-item scale where four phrases need to be set in an order of personal relevance. The CVFS assesses the 6 dimensions of the competing values framework [44]: dominant organizational characteristic, administration, management style, organizational glue, strategic emphasis and criteria for success.

Workload will be assessed with a workload questionnaire ‘werkdruklijst’ developed by De Jonge [45,46]. This scale consist of 10 items about unit workload, each item can be scored on a five-point Likert scale.

Situations, feelings and thoughts about dementia care will also be administered, with a 29-item scale, which will be published as the Strain in dementia Care (SDC) scale (Michael Bird and Anna-Karin Edberg, personal communication 2013). There’s a four-point Likert scale for each item, also a score on another four-point Likert scale can be given for professional caregiver burden related to the item. Higher scores indicate high workload.

Job satisfaction will be measured with the Maastricht Work Satisfaction Scale for Healthcare (MAS-GZ) [47]; a 21-item, five-point Likert scale that focuses on nursing staff satisfaction. It consist of seven subscales with three items each about satisfaction with: quality of care, opportunities of self-actualization/growth, supervisor, possibilities for promotion, clarity of tasks and rules, contact with colleagues and contact with patients.

‘Attitude to dementia care’ will be measured by the ADQ (see physician level) [41].

Information about demographic characteristics of the nurse will be collected: age, sex, educational level, work experience, number of years since education.

### Factors of the physical environment

Physical environmental characteristics of the DSCU will be assessed using the Therapeutic Environment Screening Survey for Nursing Homes (TESS-NH) [48]. The TESS-NH contains 84 discrete items plus an open global scale that covers 13 domains, i.e. number of patients on unit, exit control, maintenance, cleanliness, safety, orientation/cueing, privacy, unit autonomy, outdoor access, lighting, noise, visual/tactile stimulation, space/seating and familiarity/home likeliness [48].

Other information about DSCU characteristics that will be collected are: number of staff per unit, number of staff during different shifts.

### Qualitative interviews, ECP and nurse level

The ECP and 1–2 members of nursing staff will be interviewed about PDU. The qualitative interviews will be semi-structured and based on the Straussian grounded theory approach [49,50]. Interviews will be guided by a checklist of the following (relevant) topics: influence of psychosocial environment (relatives and other patients), PD prescription in practice, own beliefs, beliefs of colleagues, beliefs of patient’s family, PDU now and in the past, influence of the institution, best solutions for NPS, education, politics and media (see Table 1).

**Table 1:** Mixed methods research parameters/instruments

***Quantative***	***Parameters***	***Instruments***	***Registered by***
Patient level	Frequency of PDU	ATC classification codes	Researchers
	Appropriateness of PDU	To be announced	Researchers
	Neuropsychiatric symptoms	NPI-Q	Nurse (web based)
	Agitation and aggression	CMAI	Nurse (web based)
	Other patient characteristics	Case report file	Researchers
Physician level	Attitude to dementia care	ADQ	ECP (web based)
Nurse level	Demographic characteristics	Case report file	ECP (web based)
	Organizational culture	CVFS	Nurse (web based)
Physical environmental level.	Workload/burnout	SDC + Werkdruk (De Jonge)	Nurse (web based)
	Work satisfaction	MAS-GZ	Nurse (web based)
	Attitude to dementia care	ADQ	Nurse (web based)
	Demographic characteristics	Case report file	Nurse (web based)
	Physical environment	TESS-NH	Researchers
	Other DSCU characteristics	Case report file	Researchers
***Qualitative***			
Attitudes and beliefs	Relevant qualitative factors ECP	Semi structured interview	Researchers
	Relevant qualitative factors nurse	Semi structured interview	Researchers

Psychotropic drug use (PDU), Anatomical Therapeutical Chemical (ATC), Neuropsychiatric Inventory- Questionnaire (NPI-Q), Cohen-Mansfield Aggression Inventory (CMAI), Approaches to Dementia Questionnaire (ADQ), Elderly Care Physician (ECP), Competing Values Framework Scale (CVFS), Strain in dementia Care (SDC), the Maastricht Work Satisfaction Scale for Healthcare ’Maastrichtse Arbeidssatisfactie Schaal voor de Gezondheiszorg’ (MAS-GZ), Therapeutic Environment Screening Survey for Nursing Homes (TESS-NH).

### Data analysis

Quantitative (descriptive and multivariate) and qualitative analyses will be performed. For quantitative data analysis a multilevel model is built to investigate the potential associations with the frequency of PDU and with the appropriateness of PDU, taken into account that appropriateness of PDU is nested within DSCUs.

Data collection and analysis of the qualitative semi-structured interviews will be conducted as an iterative process with saturation as a guiding principle [51], implying interviews will be carried out until knowledge saturation is reached. This is known as the constant comparative method, which is part of the grounded theory approach [51].

### Sample size

According to the n/10 rule [52,53] 360 patients are sufficient to study the number of variables needed for this study. 67% of the patients are expected to use PDs, which means that in total 540 patients need to be recruited. Regarding good sampling and an average cluster size of 15 patients per DSCU, 36 DSCUs of twelve different nursing homes will be recruited.

### Ethical approval

The study is undertaken in accordance with the declaration of Helsinki and will be carried out in accordance with the applicable rules in the Netherlands. According to the Medical Ethics Committee of the region Arnhem-Nijmegen, the Netherlands, the study does not need to be conducted according to the Medical Research Involving Human Subjects Act (WMO), because patients will not be directly involved. Relatives, if not available other representatives, of patients will be informed and asked if they object to the collection of data. If the relatives or representatives object, patients will be excluded from the data collection.

## Discussion

The high rates of long-term PDU [9] in combination with the risk of major and hazardous side effects, limited evidence for efficacy, long-term inefficacy [15,21,22] and guidelines recommending to regularly evaluate PDU [23], make it crucial to study PDU appropriateness and its associations.

It is hypothesized that the frequency as well as appropriateness of PDU varies between DSCUs, because of factors related to patient, ECP, nurse and physical environment, as described in a conceptual framework (Figure 1). More specifically, it is expected that factors like workload and staff distress influence the appropriateness of PDU.

A strength of this study is that the recruitment focuses on nursing homes/ DSCUs with low versus those with high PDU. Knowledge about extreme, i.e. low or high, PDU and its associations is most important in dementia care. Although the instrument used for measuring appropriateness of PDU needs to be developed specifically for this study, no other instruments known are suitable to investigate the appropriateness of PDU for NPS. However, it should be taken into account that the instruments’ assessment of appropriateness of PDU relies on medical files, which may be subjected to bad reporting. Yet, in our view this procedure is considered to be more objective than personal reports of ECPs.

Many of the instruments used for this study are well known in this field of research, and will contribute to giving clear insight in factors related to PDU, which can be used in improving nursing home patient care.

The mixed design of the study is another strength of this study, interviewing ECPs and nurses can reveal relevant factors that are not measured with quantitative instruments. So, this study not only gives insight into frequency and appropriateness of PDU, but also into a diversity of possible associations, which can be used in future quantitative research.

PROPER I will provide insight in associations of (appropriateness of) PDU and thus the barriers of optimal prescription, which is the first step towards safer PDU.

## Competing interests

The authors declare that they have no competing interests.

## Authors’ contributions

SZ designed the study, DG and MS co-designed, and RK assisted in designing the study. KS wrote the paper, and DG, MS, MN, RW, CS, SZ, and RK co-wrote the paper. All authors read and approved the manuscript.

## Pre-publication history

The pre-publication history for this paper can be accessed here:

http://www.biomedcentral.com/1471-244X/13/307/prepub
